# Comparative study of caregiver stress between patients of obsessive compulsive disorder and chronic medical illness, without any physical disability

**DOI:** 10.1192/j.eurpsy.2021.1967

**Published:** 2021-08-13

**Authors:** G. Gupta, S. Nagendran

**Affiliations:** 1 Psychiatry, Teerthanker Mahaveer Medical College And Research Centre, Moradabad, India; 2 Psychiatry, Teerthanker mahaveer medical College and research centre, Moradabad, India

**Keywords:** obsessive compulsive disorder, physical medical illness, Yale-brown obsessive compulsive scale, caregiver strain index

## Abstract

**Introduction:**

Obsessive compulsive disorder (OCD) is a disabling condition that affects the quality of life of both the patient and the caregivers. Similarly, in patients with physical medical illness, caregivers face a significant amount of stress.

**Objectives:**

This study aimed to assess and compare the caregiver strain index between patients of OCD and medical illness. Moreover, this study will also compare the care giver strain index in the patients of OCD and physical medical illness depending on the severity and duration of the illness.

**Methods:**

Study was done at Department of psychiatry, Teerthanker Mahaveer University, Moradabad. In this Cross-sectional study 2 groups of caregivers were included. The group 1 included 30 caregivers of obsessive compulsive disorder patients and group 2 included 30 caregivers for physical medical illness. The Yale-Brown Obsessive Compulsive Scale was used for measuring the severity of OCD and the stress in caregivers were drawn from Caregiver strain index.

**Results:**

This study reported a high objective burden among caregivers of OCD compared with the physical medical illness (P-value=0.002). The age of the caregivers also showed to be significantly associated with the stress in both the groups. The severity of the OCD was shown to be correlated well with the stress of the caregivers (P-value=0.032). In contrast, in physical medical illness the duration of the disease showed no significant association with the caregiver’s stress.

**Conclusions:**

This study showed that in patients with OCD caregivers face a higher strain compared with the physical medical illness.

**Disclosure:**

No significant relationships.

